# Evaluating the utility of an abbreviated Consolidated Framework for Implementation Research (CFIR) for rapid qualitative analysis: a suicide prevention program case study

**DOI:** 10.1186/s43058-026-00956-5

**Published:** 2026-05-12

**Authors:** Ariel M. Domlyn, Jessica Dodge, Paul N. Pfeiffer, Claire H. Robinson, Lacey Evans, Laura J. Damschroder, Madison A. Stewart, Brittani Garlick, Jeremy B. Sussman, Andrea L. Nevedal

**Affiliations:** 1 VA Center for Clinical Management Research, VA Ann Arbor Healthcare System, P.O. Box 130170, Ann Arbor, MI 48113-0170 USA; 2 Center for Healthcare Evaluation, Research, and Promotion, Veterans Affairs Pittsburgh Healthcare System, University Drive (151C), Building 30, Pittsburgh, PA 15240 USA; 3 Department of Psychiatry, University of Michigan Medical School, 1500 E Medical Center Dr, Ann Arbor, MI 48109 USA; 4 Implementation Pathways, LLC, Chelsea, USA; 5 Department of Internal Medicine, University of Michigan Medical School, 1500 E Medical Center Dr, Ann Arbor, MI 48109 USA; 6 CUH/UCC Cancer Centre, Cork University Hospital, Cork, Ireland; 7 Cancer Research @ UCC, University College Cork, Cork, Ireland

**Keywords:** Consolidated Framework for Implementation Research (CFIR), Rapid qualitative analysis, The Pragmatic Context Assessment Tool (pCAT), Suicide prevention

## Abstract

**Background:**

The Consolidated Framework for Implementation Research (CFIR) is widely used and comprehensive. Yet the number of constructs can overwhelm users and slow qualitative analysis. The Pragmatic Context Assessment Tool (pCAT), an abbreviated CFIR-derived instrument with 14 items, may offer a streamlined approach for rapid qualitative analysis. However, the adequacy of this abbreviated tool for capturing key implementation determinants across different contexts remains unexplored.

**Methods:**

This study assessed whether the pCAT-derived CFIR constructs adequately capture implementation determinants, using a suicide prevention program case study. Semi-structured interviews (*n* = 16) were conducted across four VA medical centers with frontline mental health providers about the Suicide Prevention 2.0 Clinical Telehealth program (SP 2.0). Using methodological triangulation, we compared rapid directed content analysis using the 14 pCAT-derived constructs versus the full CFIR. The team summarized each interview using a note template that was organized by site. First, we reviewed the interview notes and used an inductive approach to identify barriers and facilitators (determinants). Determinants were copied into a matrix. Second, we conducted deductive coding of the determinants using the abbreviated pCAT construct list. Third, we expanded coding to the full CFIR when determinants did not fit the limited pCAT set. We used a consensus-based process to identify anticipated determinants and finalize the coding.

**Results:**

Of the 14 initial pCAT constructs, 11 were identified in the SP 2.0 dataset, with six representing major cross-site determinants. However, 12 additional constructs from the full CFIR were needed to comprehensively capture all barriers and facilitators, resulting in 23 total relevant constructs. Three pCAT constructs were not identified as relevant determinants. Major facilitators included Innovation Relative Advantage, Communications, Compatibility, and Innovation Deliverer Motivation. Key barriers encompassed Structural Characteristics (work infrastructure), Innovation Deliverer Capability, Reflecting & Evaluating, and Innovation Design. Most added constructs (8 of 12) belonged to the updated CFIR’s Individual Roles and Characteristics domain.

**Conclusion:**

The pCAT provides a useful starting point for rapid CFIR analysis when contexts are similar to its original development, capturing most relevant constructs while requiring significantly less analytical resources. However, the abbreviated approach provides incomplete assessment compared to full CFIR analysis. We recommend using pCAT for rapid implementation practice assessments when timely, high level results are prioritized over comprehensiveness but utilizing full CFIR when thorough determinant evaluation is paramount for research purposes.

**Supplementary Information:**

The online version contains supplementary material available at 10.1186/s43058-026-00956-5.

**Table:** Contributions to the literature

• Testing a brief context assessment tool as a rapid assessment instrument to identify barriers and facilitators to implementing innovations.
• We mapped the original items of the Pragmatic Context Assessment Tool (pCAT) to the updated Consolidated Framework for Implementation Research (CFIR).
• Established the adequacy of an abbreviated CFIR tool.
• We found pros and cons with using the pCAT (e.g., most, but not all, relevant CFIR constructs are in pCAT).

## Background

**Fig. 1:**
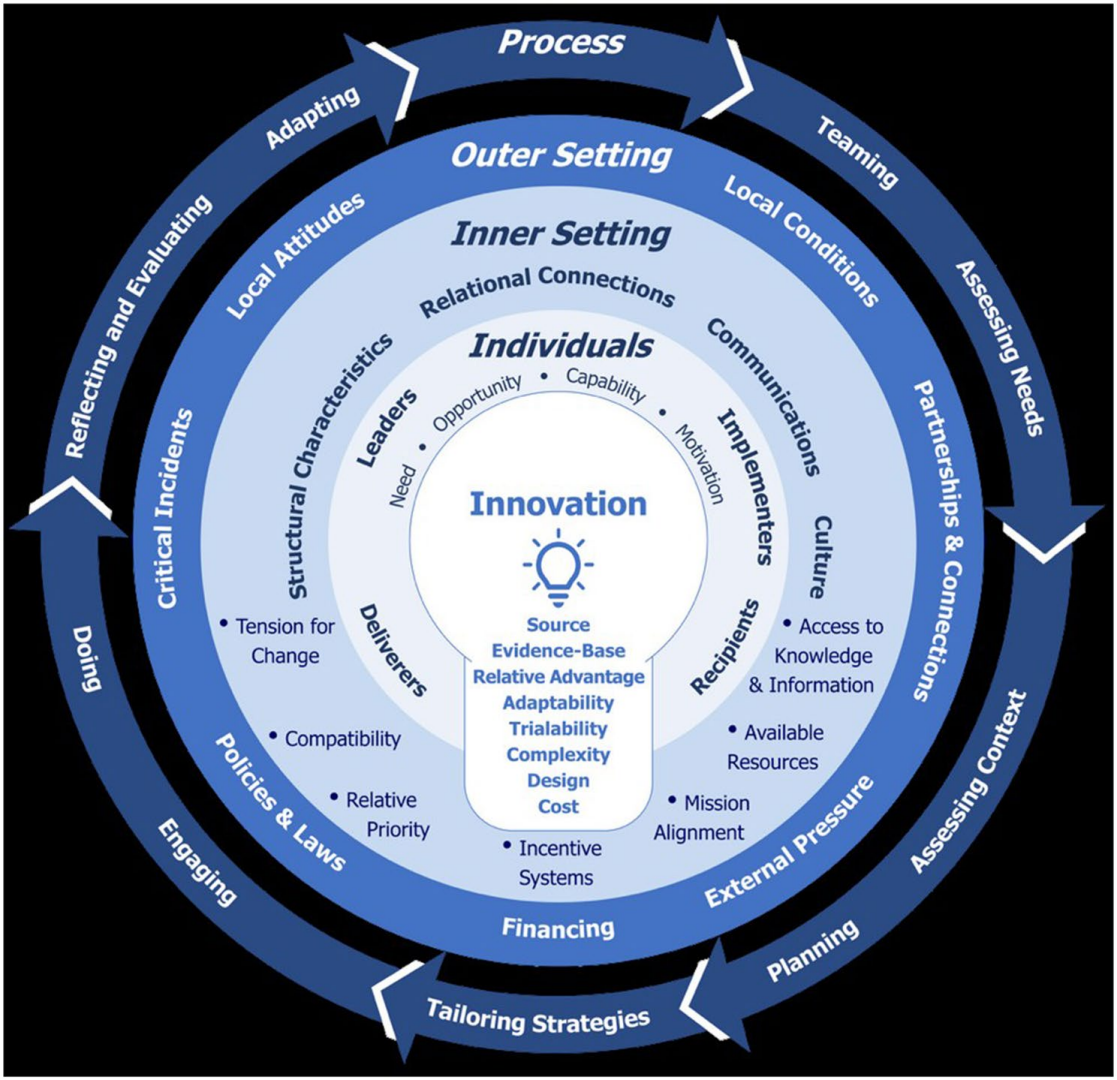
Consolidated Framework for Implementation Research (CFIR)

The Consolidated Framework for Implementation Research (CFIR) is one of the most used implementation frameworks for assessing implementation context, with over 12,000 citations within 15 years of its publication [1, 2]. First published in 2009 [3], the CFIR was updated in 2022 with a restructuring of domains and constructs based on literature review and user feedback (Fig. 1) [4]. A commonly cited reason for using CFIR is its comprehensiveness [5–8], as the framework potentially covers all known constructs affecting implementation context and process. However, its comprehensiveness – with five domains and up to 48 constructs and 19 subconstructs – can overwhelm users. Performing traditional qualitative analysis using the large number of CFIR constructs available is time-consuming [9], slowing the pace at which results are made actionable. Further, achieving competency with using CFIR is difficult: in a recent needs assessment undertaken by the CFIR Leadership Team [10], 317 implementation researchers reported needing more training to use CFIR to design an implementation evaluation (77.3%) and analyze qualitative data (71.6%). CFIR users often request support for discerning how and why to select different constructs for data collection and analysis [10]. Rapid qualitative analysis can help [9] but only if the content to be analyzed is directed and focused.

Abbreviated sets of CFIR constructs may help streamline rapid directed qualitative content analysis [9]. This is consistent with best practices in both rapid qualitative analysis and CFIR usage [10, 11] and could make implementation frameworks more user-friendly and more feasible, especially when expert qualitative implementation analysts are in short supply. However, a deductive and abbreviated suite of a priori-selected constructs may limit trustworthiness of qualitative findings [12, 13]. In this study, we assess whether selecting constructs from an existing abbreviated CFIR tool – the Pragmatic Context Assessment Tool (pCAT) [14] – for rapid analysis is adequate for capturing the breadth of key barriers and facilitators to implementing a suicide prevention program. The original goal of the pCAT development was to create a practical, easy-to-use CFIR-derived instrument for frontline teams to better understand barriers to improving a weight management program. Yet in practice the limited number of CFIR constructs contained within the pCAT are being applied for contextual assessments beyond weight management programming such as for alcohol interventions in mental health services [15] and in schools for interventions targeting physical activity [16] and behavior management [17]. Within one year (from April 2024 to April 2025), the pCAT garnered over 1,350 downloads from the CFIR website indicating an eagerness for a short CFIR-based assessment tool. Our aim was to evaluate the utility of these constructs identified by the pCAT and develop additional guidance for CFIR users.

We sought to evaluate the utility of a pCAT-derived codebook beyond the context in which the pCAT was developed. We assessed the pCAT’s adequacy for capturing context via methodological triangulation using inductive identification of barriers and facilitators then deductive coding [12]. To accomplish this, we compared a limited set of 14 CFIR constructs to using the full CFIR for rapid directed content analysis. This analysis was applied to a dataset of frontline workers in four facilities who participated in a multisite implementation project. In this paper, we describe the rationale for selecting CFIR constructs based on an existing assessment tool (the pCAT), the transformation of the interview data for rapid analysis, compare this approach to the use of the full CFIR, and provide guidance about how and when to replicate our streamlined process.

## Methods

### Setting

The US Veterans Health Administration (VA) health system is a nationwide provider of comprehensive healthcare services to Veterans. Given the high rate of suicide in this population [11], suicide prevention is a top VA priority. The Suicide Prevention 2.0 Clinical Telehealth program (SP 2.0) was launched by VA in 2021 to increase access to multimodal mental health services mitigating suicide [12]. SP 2.0 interventions include suicide safety planning, Cognitive Behavioral Therapy, Problem-Solving Therapy, and Dialectical Behavior Therapy. Clinical staff provide evidence-based treatments tailored to Veteran needs with a focus on suicide prevention. In 2023, the implementation and evaluation program Maintaining Implementation through Dynamic Adaptations (MIDAS), funded by VA’s Quality Enhancement Research Initiative (QUERI) [13], undertook a multisite project to increase SP 2.0 referrals. At the initiation of this project, the SP2.0 clinical telehealth program had largely resolved barriers related to telehealth therapist staffing and training and had established electronic medical record referral pathways. However, less than 20% of eligible VA patients with a recent documented suicide behavior were referred to the program [14], suggesting barriers at the referring provider or site level. The sampling of the four study sites was largely pragmatic and focused on VA service regions known to contain low-performing sites, though there were no specific inclusion or exclusion criteria related to baseline performance or other site characteristics.

### Data collection

MIDAS QUERI enrolled four VA medical centers which offer primary, mental health, and specialty care as well as social programs and services. Semi-structured interviews (*n* = 16) were conducted across the four participating sites. Purposive criterion sampling [15] was applied at each site to identify key informants knowledgeable about mental health service provision and suicide prevention services at their site who could provide feedback about anticipated barriers and facilitators to increasing SP 2.0 reach. The interview guide elicited local knowledge of determinants using open-ended questions; by designing interviews that did not target specific CFIR constructs, we could test our streamlined construct coding during analysis. Additional File 1 contains the semi-structured interview guide and Additional File 2 describes participant characteristics. Participants occupied different roles including suicide prevention coordinators, supervisors, psychologists, and social workers. Interviews were conducted by two qualitative analysts (CHR, LE) via Microsoft Teams. Interviews were recorded and transcribed professionally with summary notes taken by a live observer.

### Codebook development

**CFIR Construct Selection using the pCAT.** The Pragmatic Context Assessment Tool (pCAT) [14] has quickly gained popularity as an abbreviated contextual assessment. To create the pCAT, 10 constructs were selected from the 2009 CFIR based on the most commonly identified barriers across several implementation studies [18–22]: patient needs and resources, networks and communications, reflecting and evaluating, goals and feedback, compatibility, structural characteristics, available resources, tension for change, relative advantage, and leadership engagement. The pCAT questions were based on the 2009 CFIR constructs and then used to elicit responses using think-aloud interviews with frontline staff. The goal was to maximize use of lay language and minimize jargon in a short instrument. Based on participant feedback, questions were iteratively modified; the final version includes 14 items representing 10 constructs from the 2009 CFIR. For example, the item “The change is aligned with clinician values” is partially representative of the 2009 CFIR construct Compatibility. Our team used the limited set of 10 constructs within the pCAT to assess whether this subset is sufficient for use as a rapid analysis tool for implementation evaluation.

**Mapping the pCAT items to updated CFIR.** The analytic team (JD, AN, AD) mapped the 14 pCAT items, which were based on the original 2009 CFIR, to the updated CFIR. The items themselves remain unchanged, but the appropriate construct label was updated. The mapping was confirmed with CFIR developers (including LJD) and is available on cfirguide.org and in Additional File 3. Four pCAT item mappings are unchanged but the remaining 10 items were updated to align with updated CFIR. For example, the aforementioned item “The change is aligned with clinician values” was within the 2009 CFIR *Compatibility* construct, but with the updated CFIR the item now maps to the *Innovation Deliverer: Capability*. This lengthened the number of constructs within the pCAT — whereas previously the 14 items mapped to only 10 constructs, they now map to 14 updated constructs.

**Initial Codebook.** The resulting mapped list of 14 updated CFIR constructs within the pCAT is as follows: Innovation domain – Innovation Relative Advantage. Inner Setting domain – Culture: Recipient-centeredness, Communications, Mission Alignment, Compatibility, Structural Characteristics: Work infrastructure, Available Resources: Space, Available Resources: Materials and equipment, Tension for Change. Individuals domain – High-level Leaders: Motivation, Mid-level Leaders: Motivation, Innovation Deliverers: Capability, Innovation Deliverers: Opportunity. Process domain – Reflecting & Evaluating. Next, the team developed a codebook with descriptions tailored to the SP 2.0 program implementation. Notably, in this rapid analysis coding was applied after data were summarized in an Excel matrix; this differs from traditional qualitative coding.

**Final Codebook.** The codebook was iteratively refined during the rapid analytic process (see subsequent section). The distinctions between the initial and final codebooks are described in the Results section. Final SP 2.0 program codebook descriptions are in Additional File 4. Utility of the pCAT is based on methodological triangulation [16] of the difference between the initial and final codebooks.

**Fig. 2:**
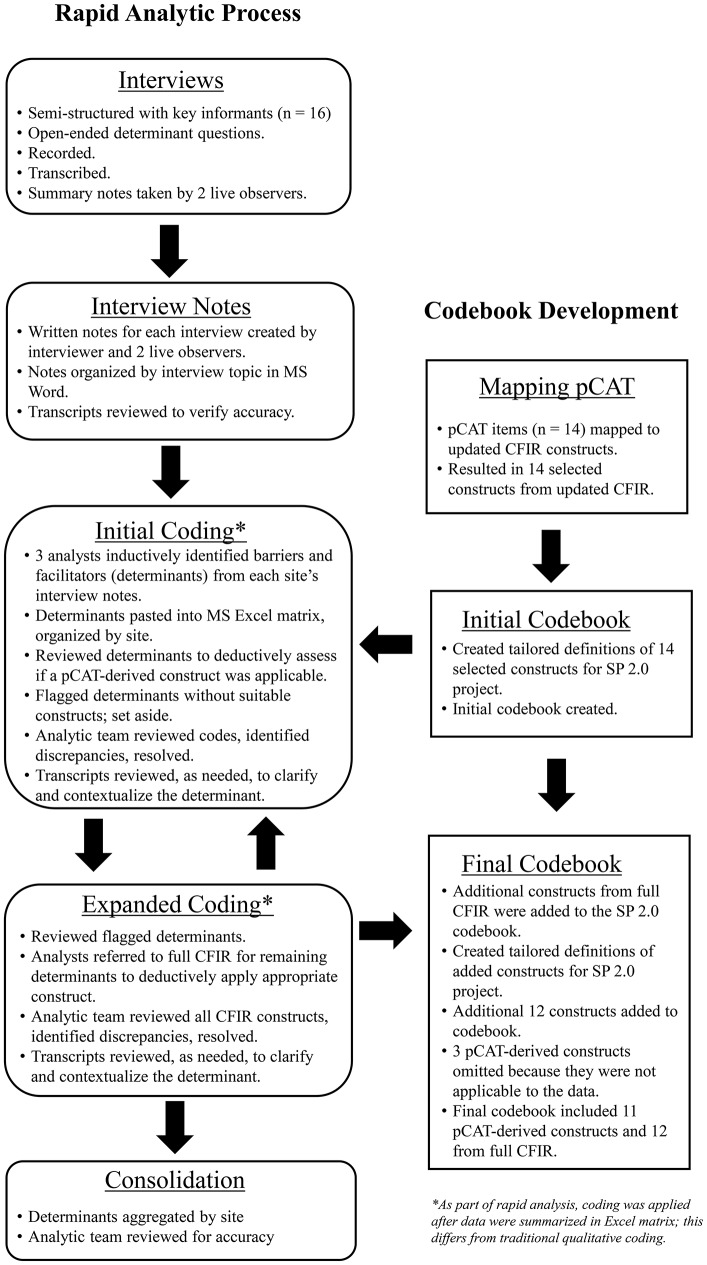
Analytic process

### Rapid analytic process

Once the subset of updated constructs was selected, we turned to SP 2.0 data analysis. We conducted rapid directed content analysis, which is a rigorous streamlined approach to qualitative analysis [9, 17, 23]. See Fig. 2.

**Interview Notes.** Observers (MS, LE, CHR) took comprehensive notes during interviews. After the interviews, the primary notetaker, secondary notetaker, and interviewer reviewed the notes and made corrections to the documents, referring to recordings as needed. Notes were organized by interview topic in MS Word. The dataset for this study focused on responses to interview questions focused on what has/has not worked well with the SP 2.0 program and questions “What do you feel are the barriers to appropriately referring to SP 2.0 Clinical Telehealth” and “What are the facilitators for a smooth referral to SP 2.0 Clinical Telehealth?”

**Initial Coding.** To identify anticipated CFIR determinants, analysts (JD, AN, AD) followed three primary steps. First, analysts reviewed each set of interview notes to inductively identify the SP 2.0 referral barriers and facilitators (determinants). These determinants were entered into an MS Excel matrix, organized by interview, then aggregated for each site. Second, the analysts deductively coded the determinants using the 14 pCAT-derived CFIR constructs. Third, the team reviewed each site-level cluster of determinants together to discuss and confirm the appropriateness of the CFIR constructs from the initial codebook, which was limited to the 14 pCAT-derived constructs. Some determinants remained uncoded due to no appropriate construct found in the initial codebook or were flagged as a partial match to be reviewed in the next step. We used the first six interview note documents as an analytic training dataset and used a consensus-based process to finalize the approach inductively identifying determinants and deductively applying codes. As needed, the team referred to full transcripts.

**Expanded coding.** In instances where one of the 14 pCAT-derived constructs was not applicable to a particular barrier or facilitator (determinant), we then referred to the full list of constructs in the updated CFIR and deductively coded using the appropriate construct. Once we established consistency via the training dataset, we assigned a primary and secondary analyst to identify barriers and facilitators in the remaining summaries and code for the relevant CFIR constructs. We continued to meet weekly to discuss discrepancies in our analysis. We collectively reviewed instances where a determinant did not fit a CFIR construct from the abbreviated construct list, then applied the most appropriate construct from the full updated CFIR to develop the final codebook.

**Consolidation.** The site-level barriers and facilitators were summarized and presented to suicide prevention partners who oversee VA programs and administer SP 2.0 nationally.

## Results

In the following sections we first provide a high-level summary of CFIR determinants that may impact future scale-up of SP 2.0 and then describe results of applying the streamlined CFIR constructs. CFIR constructs are italicized and constructs that were not part of the initial 14 constructs – as derived from the pCAT – are noted with an asterisk in the text below.

### Project findings: pre-implementation determinants of SP 2.0

The four major cross-site facilitators were *Innovation Relative Advantage* of the SP 2.0 program (belief that remote psychotherapy care can better reach Veterans at high risk for suicide), *Communications* (effective and timely communication with the SP 2.0 resource hub), *Compatibility* (referral process to SP 2.0 fit well with clinical routines), and Innovation Deliverer: Motivation* (providers felt optimistic about providing SP 2.0 to at-risk Veterans). The four major cross-site barriers were *Structural Characteristics: Work Infrastructure* (high staff turnover, which decreased institutional knowledge about the program), *Innovation Deliverer: Capability* (lack of provider knowledge and education about SP 2.0 offerings and referral process), *Reflecting & Evaluating* (unknown how or whether the facility monitors or tracks a Veteran after SP 2.0 referral), and Innovation Design* (insufficient material to explain SP 2.0 eligibility criteria). The major cross-site determinants are bolded in Table 1. Other determinants appeared in the data but were not consistently identified across sites.

**Table 1:** CFIR constructs in initial and final codebooks

Updated CFIR Domains	Initial Codebook: Constructs in pCAT	Final Codebook: Constructs in SP 2.0 Codebook
Innovation	Innovation Relative Advantage	**Innovation Relative Advantage**
Individuals	Innovation Deliverers: Capability	**Innovation Deliverers: Capability**
	Innovation Deliverers: Opportunity	Innovation Deliverers: Opportunity
	High-level Leaders: Motivation	High-level Leaders: Motivation
	Mid-level Leaders: Motivation	Mid-level Leaders: Motivation
Inner Setting	Compatibility	**Compatibility**
	Communications	**Communications**
	Structural Characteristics: Work Infrastructure	**Structural Characteristics: Work Infrastructure**
	Tension for Change	Tension for Change
	Mission Alignment	Mission Alignment
	Culture: Recipient-Centeredness	
	Available Resources: Space	
	Available Resources: Materials & Equipment	
Process	Reflecting & Evaluating	**Reflecting & Evaluating**
Innovation		**Innovation Design**
Individuals		High-level Leaders: Capability
		High-level Leaders: Opportunity
		Mid-level Leaders: Capability
		Mid-level Leaders: Opportunity
		**Innovation Deliverers: Motivation**
		Innovation Recipients: Capability
		Innovation Recipient: Opportunity
		Innovation Recipient: Motivation
Inner Setting		Relative Priority
		Structural Characteristics: Information Technology Infrastructure
		Access to Knowledge & Information

Bold indicates major cross-site determinant

Analysis was completed after the MIDAS QUERI team began their SP 2.0 optimization project. Results were shared with the SP 2.0 operational partners to improve their implementation planning. Site-specific determinants were described using case examples and illustrative quotes. The SP 2.0 program leadership acknowledged awareness of some barriers and described efforts to improve them, including clarifying inclusion criteria and increasing education and outreach to facilities, particularly to suicide prevention coordinators.

### Methodological findings: process utility

**Initial vs. final codebook.** Although we initially selected 14 CFIR constructs from the pCAT mapping, during analysis we identified instances where the barrier or facilitator did not appropriately match any constructs within that subset. In addition to the initial 14 CFIR constructs from the pCAT, appropriate determinant labels were identified from the full CFIR resulting in adding 12 more constructs to comprehensively identify the anticipated barriers and facilitators to SP 2.0 implementation (Table 1). Further, three of the 14 initial constructs were *not* identified as anticipated determinants in the SP 2.0 project data, all of which were from the Inner Setting domain – *Culture: Recipient-Centeredness*, *Available Resources: Space*, *Available Resources: Materials & Equipment*. Overall, we identified 23 updated CFIR constructs that were relevant to anticipated barriers and facilitators to SP 2.0 program implementation in at least one VA site: 11 within pCAT and 12 added from full CFIR. These included constructs from four of the five updated CFIR domains: Innovation, Inner Setting, Individuals: Roles and Characteristics, and Process. Although one pCAT item was originally mapped to an Outer Setting domain construct in the 2009 CFIR (*Patient Needs & Resources*), the updated CFIR changed that item’s mapping to an Inner Setting construct (*Culture: Recipient-centeredness*).

**Final codebook: Constructs by domain.** From the 14 constructs originally in the codebook, 11 appeared in the SP 2.0 dataset: one from the Innovation domain (*Innovation Relative Advantage*), four from the Individuals domain’s subdomains Roles & Characteristics (*Innovation Deliverers: Capability*,* Innovation Deliverers: Opportunity*,* High-level Leaders: Motivation*,* Mid-level Leaders: Motivation*), five from the Inner Setting domain (*Compatibility*, *Communications*, *Structural Characteristics: Work Infrastructure*, *Tension for Change*, *Mission Alignment*), and one from the Process domain (*Reflecting & Evaluating*). Three of the 14 initial constructs were *not* identified as determinants to the SP 2.0 project data, all of which were from the Inner Setting domain (*Culture: Recipient-Centeredness*, *Available Resources: Space*, *Available Resources: Materials & Equipment*). The analysts identified 12 additional constructs to describe barriers and facilitators to SP 2.0: one from the Innovation domain (Innovation Design*), eight from the Roles & Characteristics subdomains (High-level Leaders: Capability*, Opportunity*; Mid-level Leaders: Capability*, Opportunity*; Innovation Deliverers: Motivation*; Innovation Recipients: Capability*, Opportunity*, Motivation*) and three from the Inner Setting domain (Relative Priority*, Structural Characteristics: Information Technology Infrastructure*, Access to Knowledge and Information*). Notably, most constructs added to the final codebook were within a new domain added to the updated CFIR — Individuals: Roles and Characteristics.

## Discussion

This study is the first to use the pCAT tool to create a “shortcut” method to select CFIR constructs for rapid qualitative analysis. The SP 2.0 context was sufficiently similar to the pCAT developers’ (both in national programs in the VA with data collected from frontline providers), yet also distinct (the innovation was suicide prevention rather than weight management). The pCAT identified 14 CFIR constructs from prior projects [18–22]. Eleven of the 14 constructs were identified in the SP 2.0 project data for the initial codebook, and six of those were identified as salient cross-site determinants. Although 12 constructs were added to the final codebook, only two of those were notable across sites. This method established that using a priori construct selection from a CFIR tool is a sufficient method for rapid analysis: methodological triangulation revealed that most CFIR constructs are contained in the pCAT, despite differences in our project context from the original pCAT developers’ context. However, using the abbreviated list of constructs contained in the pCAT provided an incomplete assessment of the data. Therefore, we do not recommend relying solely on the pCAT in instances where thorough and rigorous determinant evaluation is paramount. CFIR constructs should be prioritized based on the project and definitions should be tailored to the innovation and setting [9, 10].

### Exploration of relevant and irrelevant constructs

Three constructs were selected for the initial deductive codebook but not found as relevant in the dataset; there are several potential reasons. Two of these constructs – Available Resources: Space and Available Resources: Materials & Equipment – were relevant for the innovation in which the pCAT was first developed [24, 25] but appear irrelevant for referring to a virtual program such as SP 2.0. The innovation being implemented [26] — referring appropriate patients to the SP 2.0 clinic — simply requires an electronic health record with consultation capabilities, a standard resource in all medical facilities. This highlights the need to select constructs based on the specific resource needs of the innovation itself, not on the basis of generally common determinants. The remaining construct, Culture: Recipient-Centeredness, was not mentioned by interviewees though this often is an important concept. It could be a feature of the interview pool and specific innovation being implemented. The project goal was to increase referrals to SP 2.0, not delivery of the SP 2.0 program itself. The program itself was already established and staffed as a virtual offering throughout VA, but the referrals were low. Given this goal, the interview guide gathered perceptions among the mental health providers responsible for referring patients, not perceptions among the SP 2.0 staff providing the intervention. 

One potential reason for the additional twelve constructs in the final codebook is because of a feature of updated CFIR that did not exist when the pCAT was created. The majority (eight) of the added constructs were from the Individuals: Roles and Characteristics domain that were not present in the original CFIR. For the Roles subdomain, CFIR use guidelines [10] suggest listing all actors involved in the implementation process, from the *High-level leaders* (e.g., mental health service chiefs) to *Other Implementation Support* (e.g., the SP 2.0 program leads) to *Innovation Deliverers* (e.g., mental health providers) to *Innovation Recipients* (e.g., Veterans at risk of suicide). Characteristics include four constructs: *Need* (not found in the present data), *Capability*, *Opportunity*, and *Motivation*. Each characteristic can be found in every role, resulting in myriad permutations of implementation determinants. The addition of the Characteristics subdomain in the updated CFIR comes from the well-known literature of the COM-B framework [27], which supplements the organizational focus of CFIR by adding individual-level attributes.

Another potential reason for additions to the final codebook arises from the contrast of generality versus granularity. Three constructs added to the final codebook were features of the Inner Setting with close proxies in the original codebook. For example, *Relative Priority* (an addition) is similar to *Innovation Relative Advantage* (an initial construct) and *Tension for Change* (an initial construct). Further, *Structural Characteristics: Information Technology Infrastructure* (an addition) is similar to *Structural Characteristics: Work Infrastructure* (an initial construct). Finally, *Access to Knowledge & Information* (an addition) is similar to *Innovation Deliverers: Capability* (an initial construct). These additions may be reflective of the analyst team’s interest in strong CFIR specificity and accuracy to inform implementation research and, thus, may not indicate adequacy of the process. The utility of methodological simplicity versus accuracy is described further below. This methodological work illustrates one example of how implementation science can be beneficial to suicide prevention (Larkin et al., 2023). Contextualizing our results for suicide prevention programming, several findings are apparent. The *Innovation Relative Advantage* is key to adoption of new suicide prevention protocols, in support of prior findings that structure suicide interventions are perceived more positively than ad hoc assessment or existing tools [28]. The addition of Individuals: Roles and Characteristics domain for our methods also aligns with systematic review findings that *Innovation Deliverers: Motivation* and *Innovation Deliverers: Capability* are salient implementation determinants [29]. However, our work adds *Innovation Deliverer: Opportunity* as a potential determinant for consideration in suicide prevention referral. Identifying suicide prevention barriers such as these are critical for effectively improving timely access to intervention [30] .

### Recommendations

We have several suggestions for analysts creating a deductive codebook using a priori implementation constructs from CFIR. First, during data collection we reiterate the guidance [10] to incorporate open-ended questions—in addition to construct-targeted questions—to capture determinants that may otherwise be missed. Second, during analysis, analysts should plan for additional inductive coding using CFIR [4]. These inductive codes can be later mapped to the same determinant framework used for a priori data collection design. Third, when using determinants from previous works to design either data collection or analytic plans, we recommend finding either published studies or preliminary evidence that are closely analogous in setting, innovation, and respondent type. An example from prior work [18] interviewed operational leaders to identify an initial set of high-priority constructs, then iteratively refined the list with key informants. Fourth, we also recommend using theory, not solely a framework, to identify constructs. The distinction between implementation frameworks and theories is described elsewhere [19, 20], and we will reiterate the importance of using theory to guide construct selection for data collection and analysis. CFIR is a framework, not a theory, because it does not ascribe relationships between the constructs [4] and focuses on form over function. Understanding complex relationships and causality between determinants is the purview of existing theories and observational theorizing as a form of analysis [21, 22]. 

Fifth, methods should be scaled to project needs. An abbreviated deductive codebook—such as we derived from the pCAT—may be most helpful in implementation practice (i.e., not science), when the analyst needs to quickly relay information to partners but not assume transferability. Drawbacks to this approach are that faster is not always better [31] and may limit the ability to develop micro-theories [32]. Further, if that work is published then the authors must be clear that determinant specificity was sacrificed for the sake of rapid translation. As we continue building the science of implementation and finding balance between rigor and feasibility [31], the field must be clear about which determinant studies are useful for syntheses to build grand theories [32] and which studies solely served the need for that specific setting or partner. For the latter, it may be sufficient to use the process we outlined.

Finally, using the full CFIR as an a priori codebook is often not practical or necessary, and is reserved for instances where the primary research aim is to answer implementation methodological questions, for example in hybrid III projects [24, 25] or when studying causal implementation mechanisms [26, 27]. In those instances, we recommend relying on a qualitative analyst with formal implementation science training or extensive experience in implementation research. To ease the process, rather than line coding to each construct, the analyst can summarize the barriers and facilitators by case—or develop a micro-theory [22]—and then apply the codes to summaries. It is not necessary to summarize barriers first, but it may reduce the amount of time compared to line by line coding with the full CFIR. Coupling this summarize-first process with rapid qualitative analysis, as demonstrated here, can speed the translation of context into actionable information for improving implementation process without compromising rigor. Rapid analysis is timely and reliable [17, 23, 35, 36], particularly for CFIR [9]. Resources are available for the appropriate planning and reporting of rapid qualitative research [17].

### Limitations

Our methodology applied an abbreviated codebook to pre-implementation data. Although we found this codebook incomplete and added more determinant codes, our results are limited because we did not do a pre/post analysis. Possibly, the original abbreviated codebook was sufficient to identify the determinants most predictive of implementation success. This needs further study using a more robust dataset. Further, we did not follow our own recommendation of using theory to select constructs. This was intentional, in reaction to CFIR users’ high interest in the pCAT tool, desire for additional information about CFIR analytic methods, and proliferation of studies using solely frameworks for construct selection. As a result, we cannot contrast this method with others for a priori codebook creation. Another limitation is that “the thing” [32]in this study was referrals to SP 2.0, not the SP 2.0 program itself. Inherently referral pathways will result in greater focus on inner setting barriers than other innovations. Further, the informant sample of suicide prevention coordinators and VA mental health clinicians did not raise issues determinants such as risk assessment stigma (Outer Setting determinants) due to their roles; this may differ in other suicide prevention programs. Finally, our results are limited to CFIR use and may not yield similar results for other implementation frameworks. Again, this was intentional due to the widespread use of CFIR in research and practice. Additional guidance for selecting CFIR constructs can be found elsewhere [10] and will continue to be developed.

## Conclusion

One of our goals was to assess whether the 14 constructs within the pCAT could be used as a rapid CFIR construct selection tool. The pCAT was created for a very specific need: to be used within healthcare systems (not settings like schools or community-based agencies), solely when frontline clinicians are the sample (as it did not include administrators, leadership, or other roles as recommended in implementation research), and for conducting a quality improvement study (not implementing a new program). Therefore, the pCAT can be a starting point for identifying potential determinants when there is strong overlap between those conditions but should not be the sole method used. The pCAT may also be appropriate for teams without deep CFIR methodological knowledge because it allows for a high-level assessment of contextual barriers using a more concise set of constructs. In these instances, we stress the need to acknowledge and report the limitations of this method. In summary: tools such as the pCAT are appropriate assessment instruments for specific contexts, such as when rapid information translation is a higher goal than generalizability or building research knowledge. When comprehensiveness and specificity are the goals, determinant selection should follow established guidelines [10].

## Electronic Supplementary Material

Below is the link to the electronic supplementary material.

Supplementary Material 1: Additional File 1. SP 2.0 MIDAS Interview Guide. Additional File 2. Participant Characteristics. Additional File 3. The pCAT items mapped to the updated CFIR. Additional File 4. SP 2.0 Program Codebook

## Data Availability

The datasets used during the current study are not available for public dissemination due to regulatory guidelines.

## Abbreviations

CFIR: Consolidated Framework for Implementation Research
MIDAS: Maintaining Implementation through Dynamic Adaptations
pCAT: Pragmatic Context Assessment Tool
QUERI: Quality Enhancement Research Initiative
SP 2.0: Suicide Prevention 2.0 program
VA: U.S. Department of Veterans Affairs
