# Supplementary material for: Evaluating the utility of an abbreviated Consolidated Framework for Implementation Research (CFIR) for rapid qualitative analysis: a suicide prevention program case study
Source: Implement Sci Commun. 2026 May 12;7:129. doi: 10.1186/s43058-026-00956-5 (PMC13352689; doi:10.1186/s43058-026-00956-5)
Supplement: Supplementary file 1 — Supplementary Material 1: Additional File 1. SP 2.0 MIDAS Interview Guide. Additional File 2. Participant Characteristics. Additional File 3. The pCAT items mapped to the updated CFIR. Additional File 4. SP 2.0 Program Codebook [file 43058_2026_956_MOESM1_ESM.docx]

**Additional Files**

Additional File 1 for “Evaluating the utility of an abbreviated Consolidated Framework for Implementation Research (CFIR) for rapid qualitative analysis: A suicide prevention program case study.”

**MIDAS SP 2.0 Clinical Telehealth Interview Guide**

Domlyn et al., Applying an abbreviated Consolidated Framework for Implementation Research (CFIR) with rapid qualitative analysis: Pre-implementation Assessment of a suicide prevention program.

My name is [interviewer name] and I am from the VA in Ann Arbor, Michigan. With me are [2^nd^ interviewer name] and [note taker name]. We are part of the MIDAS QUERI team made up of researchers and physicians based at the Center for Clinical Management Research at the Ann Arbor VA. MIDAS is a quality improvement program focused on increasing appropriate use of SP 2.0 Clinical Telehealth by decreasing the variability in implementation of evidence-based practice. We’re interested in hearing your thoughts about barriers and facilitators to appropriate use of SP 2.0 Clinical Telehealth.

*Language clarification at outset*: The SP 2.0 program accepts referrals from patients with suicidal self-directed violence or preparatory behaviors in the past 12 months. Hereafter we will refer to this as “a recent suicide attempt.”

Taking part in this interview is completely voluntary. Anonymity and confidentiality of participants will be preserved by limiting access to identifiable information to only our project team. Findings shared outside of our team will be de-identified and reported in aggregate at the facility level.

The interview will last no longer than 1 hour. You can skip any questions you prefer not to answer. You can stop the interview at any time. We would like to record this interview so that we have a complete and accurate record of the information you provide. You may ask to pause or stop recording at any time. The audio file will be stored in a restricted access file on a secure VA server with access limited to a select set of team members.

Do I have your permission to record the interview?

Do you have any questions before we get started?

[Turn on recorder] Okay, to confirm, I’m starting the recording. Is this ok with you?

1. Please tell me your title and role in VA.
   1. How long have you been in this position?
   2. How long have you worked at this facility? (Where did you work prior?)
2. What is your experience to date with SP 2.0 Clinical Telehealth?
   1. What has worked well?
   2. What about not as well?
   3. What have been the outcomes of your referrals to the service? [Were your consults accepted? Did patients get seen quickly? Did they complete treatment? Did it help them?]
   4. What feedback do you receive from the SP 2.0 Clinical Telehealth staff about your referrals?
   5. What feedback do you receive from the patients you refer to SP 2.0 Clinical Telehealth?
   6. For Suicide Prevention Coordinators: What is it like to work with the Clinical Resource Hub?
      1. Clinically?
      2. Administratively?
3. Can you describe SP 2.0 Clinical Telehealth referral process at your facility? How does it fit into your workflow?
   1. Who is responsible for making SP 2.0 Clinical Telehealth referrals? (e.g., the provider who first hears about the attempt, a centralized referral contact, etc.)
      1. Which patients are appropriate for referral?
      2. What modifications, if any, would you make to the referral criteria?
   2. What data sources and/or metrics does your facility use to monitor suicide attempts and the care patients receive after a suicide attempt?
   3. How has the SP 2.0 Clinical Telehealth referral process changed, if at all? Why? (e.g., root cause analysis, sentinel event, quality improvement)
4. How important is it to you that patients receive an evidence-based psychotherapy specific for suicide prevention?
5. What do you feel are the barriers to appropriately referring to SP 2.0 Clinical Telehealth?
   1. What are the facilitators for a smooth referral to SP 2.0 Clinical Telehealth?
   2. What are your thoughts about managing suicide prevention locally versus through a centralized telehealth program?
   3. To what extent do you participate in improving use of SP 2.0 Clinical Telehealth?
   4. If suicide prevention is managed to any degree locally, what does that look like?
      1. Who is involved?
6. How important is increasing referrals to/appropriately utilizing SP 2.0 Clinical Telehealth to you compared to other priorities?
   1. How important is increasing referrals to local leadership?
   2. How important to patients are the services offered by SP 2.0 Clinical telehealth?
7. What has your experience with academic detailing been?
   1. Quality/Process Improvement?
   2. Lean?
   3. How do you find out about new clinical processes/approaches/evidence related to suicide prevention?
8. What are your recommendations for how to optimize use of SP 2.0 Clinical Telehealth?
   1. How could people in different roles (e.g., nurses, medical assistants, pharmacists, administrative, etc.) help?
   2. How do you feel academic detailing might help?
   3. How do you feel about an academic detailer who is not a VA mental health clinician?
9. Is there anything I haven’t asked about today that you feel is important to mention with regards to appropriate use of SP 2.0 Clinical Telehealth?
10. We define fully “successful implementation” as all Veterans with a recent suicide attempt being considered for SP2.0 clinical telehealth and all those who are appropriate (e.g., are eligible and agree to participate) being referred to the program. With that in mind: Overall, from a scale of 1 to 10, where 1 is unsuccessful, and 10 is fully successful, how successful will you/your facility be in implementing referrals to SP2.0 clinical telehealth?
    1. Why did you choose that number?
    2. What would it take to increase that number.

Thank you very much for taking the time to participate in this interview. Your thoughts and insights have been immensely helpful and will be incorporated into our work as we continue to focus on decreasing variability in the implementation of evidence-based practices designed to facilitate appropriate use of SP 2.0 Clinical Telehealth.

Additional File 2 for “Evaluating the utility of an abbreviated Consolidated Framework for Implementation Research (CFIR) for rapid qualitative analysis: A suicide prevention program case study.”

Table S2. Participant Characteristics

| **Site** | **ID** | **Role** |
| --- | --- | --- |
| A VAMC in VISN 4 | 4000 | Suicide Prevention Program Supervisor |
|  | 4001 | SPC/Suicide Postvention Team Lead |
|  | 4002 | Outpatient Section Chief |
|  | 4003 | SPC/REACH VET Coordinator |
|  | 4005 | Social Worker/Program Coordinator |
|  | 4006 | Clinical Psychologist |
| A VAMC in VISN 18 | 4007 | Clinical Psychologist |
|  | 4008 | Psychologist |
|  | 4010 | SPC |
|  | 4021 | General Mental Health section chief |
| A VAMC in VISN 17 | 4027 | Clinical Social Worker/SPC |
|  | 4057 | Chief of Social Work |
|  | 4063 | Clinical Psychologist |
| A VAMC in VISN 4 | 4058 | Suicide Prevention Case Manager |
|  | 4064 | Licensed Professional Mental Health Counseling Supervisor |
|  | 4065 | Psychiatric Nurse Practitioner |

Additional File 3 for “Evaluating the utility of an abbreviated Consolidated Framework for Implementation Research (CFIR) for rapid qualitative analysis: A suicide prevention program case study.”

Table S3. pCAT items mapped to updated CFIR constructs

| **pCAT item** | **2022 updated CFIR construct** |
| --- | --- |
| People here regularly seek to understand the needs of patients and make changes to better meet those needs. | Culture: Recipient-Centeredness |
| I have open lines of communication with everyone needed to make the change. | Communications |
| I have access to data to help track changes in outcomes. | Reflecting & Evaluating |
| The change is aligned with leadership goals. | Mission Alignment |
| The change is aligned with clinician values. | Innovation Deliverer: Capability |
| The change is compatible with existing clinical processes. | Compatibility |
| The structures and policies in place here enable us to make the change. | Structural Characteristics: Work Infrastructure |
| We have sufficient space to accommodate the change. | Available Resources: Space |
| We have sufficient time dedicated to make the change. | Innovation Deliverer: Opportunity |
| We have other needed resources to make the change (staff, money, supplies, etc.). | Available Resources: Materials & Equipment, Funding |
| People here see the current situation as intolerable and that the change is needed. | Tension for Change |
| People here see the advantage of implementing this change versus an alternative change. | Relative Advantage |
| Higher level leaders are committed, involved, and accountable for the planned improvement. | High-level Leaders: Motivation |
| Leaders I work with most closely are committed, involved, and accountable for the planned improvement. | Mid-level Leaders: Motivation |

Additional File 4 for “Evaluating the utility of an abbreviated Consolidated Framework for Implementation Research (CFIR) for rapid qualitative analysis: A suicide prevention program case study.”

Table S4. SP 2.0 Rapid Analysis Codebook

| **In the original pCAT?** | **CFIR Construct** | **CFIR Construct Standard Definitions** | **Construct SP 2.0-tailored definition and coding guidance** |
| --- | --- | --- | --- |
|  | **I. INNOVATION DOMAIN** | The “thing” being implemented (20), e.g., a new clinical treatment, educational program, or city service. | SP 2.0 Description: Suicide Prevention 2.0 Clinical Telehealth is a recent VA initiative that offers virtual evidence-based treatment to Veterans with recent suicidal self-directed harm behavior. |
| No, added in because was relevant to SP 2.0 | Innovation; Design | The innovation is well designed and packaged, including how it is assembled, bundled, and presented. | The extent to which SP 2.0 is well designed and packaged. Interview participants' views regarding if there is accessible information about the program that can be easily delivered to mental health clinicians and coordinators as well as other clinical leadership to disseminate and educate staff about general SP 2.0 information, eligibility for the SP 2.0 program, the referral process, and likelihood of positive Veteran outcomes. |
| Yes | C. Innovation Relative Advantage | The innovation is better than other available innovations, or current practice. | Do informants believe SP 2.0 (as a virtual VISN level treatment option) is a better treatment option than other mental health treatments available to Veterans with a recent self-directed harm incident? (e.g., SP 2.0 compared to "in house" mental health treatment options such as for depression or anxiety) |
|  | **II. OUTER SETTING DOMAIN** | Define the Outer Setting(s): The setting outside the facility, i.e., VISN + VA and as well as the community/state/country. | Veteran Integrated Service Network (VISN) (18 regions across the nation); national Veteran Health Administration policies |
|  | **III. INNER SETTING DOMAIN** | Define the Inner Setting(s): The facility | The clinic level (i.e., mental health clinic, or substance use clinic), local hospital policies |
| No, added in because was relevant to SP 2.0 | A2. Structural Characteristics: Information Technology Infrastructure | Technological systems for tele-communication, electronic documentation, data storage, management, reporting, and analysis; Supports functional performance in the Inner Setting. | The extent to which informants believe there are technological systems in place for routine mental health operations (e.g., electronic consult/referral process, potential for reminders in the medical record system) |
| Yes | A3. Structural Characteristics: Work Infrastructure | Organization of tasks and responsibilities, within and between individuals and teams; Supports functional performance in the Inner Setting. | The extent to which informants believe there are appropriate staffing at the facility to maintain mental health operations, such as, designated staff responsibilities and workload of staff and communication across disciplines/lines of service (i.e., Emergency department to mental health service) |
| No, added in because was relevant to SP 2.0 | C. Communications | There are high quality formal and informal information sharing practices within and across Inner Setting boundaries (e.g., structural, professional). | There are high quality formal and informal information sharing practices within and across Inner Setting boundaries (e.g., structural, professional). |
| Yes | E. Tension for Change | The current situation is intolerable and needs to change. | The extent to which informants believe the current state of suicide prevention programs are not meeting the needs of Veterans at high risk for suicide |
| Yes | F. Compatibility | The innovation fits with workflows, systems, and processes. | How SP 2.0 fits within existing mental health treatment and care coordination processes. |
| No, added in because was relevant to SP 2.0 | G. Relative Priority | The degree to which implementing and delivering the innovation is important compared to other initiatives | The extent to which referring to SP 2.0 is an important priority over other initiatives. Note: This is not capturing if clinicians think Veterans who are at high risk for suicide should be referred to mental health programs, it is capturing the relative importance of the SP 2.0 program in comparison to other mental health treatments and programs that are currently offered to Veterans at high risk for suicide. |
| Yes | I. Mission Alignment | Implementing and delivering the innovation is in line with the overarching commitment, purpose, or goals of the Inner Setting. | SP 2.0 goals are aligned with the facility's mission and purpose for Veteran mental healthcare. |
| No, added in because was relevant to SP 2.0 | K. Access to Knowledge | Guidance and/or training is accessible to implement and deliver the innovation. | Informants report knowledge (or lack of knowledge) of educational information pertaining to SP 2.0 |
|  | **IV. INDIVIDUALS DOMAIN** | Perceptions of ***Individuals***, across different roles and characteristics. Note: These may be self-perceptions for some roles. | Participants perceptions of others or themselves re: characteristics of the individuals that were important to the implementation of SP 2.0 (i.e., education, referrals) |
|  | **ROLES x CHARACTERISTICS SUBDOMAIN** | Define Roles: Suicide Prevention Coordinators, Suicide Prevention Supervisors, Mental Health Clinicians (LMSWs, clinical psychologists), Mental Health Treatment Coordinators, | Define Roles: Suicide Prevention Coordinators, Suicide Prevention Supervisors, Mental Health Clinicians (LMSWs, clinical psychologists), Mental Health Treatment Coordinators, |
| Yes | A. High-Level Leaders | Individuals with a high level of authority, including key decision-makers, executive leaders, or directors | National/VISN/Facility director. General feedback about the Clinical directors/chiefs or hospital leadership. Note: if the participant is in this role or speaking about others |
| Yes | High level leaders: Capability | The individual(s) has interpersonal competence, knowledge, and skills to fulfill Role (*of referral to SP 2.0*). | The individual has sufficient understanding of SP 2.0 to effectively educate staff and support staff to refer to SP 2.0 |
| Yes | High level leaders: Opportunity | The individual(s) has availability, scope, and power to fulfill Role (*of referral to SP 2.0*). | The individual has the time, autonomy, and power to perform their role in supporting SP 2.0 education, referral, and follow-up. Note: may also code % effort/funding/protected time to funding |
| No, added in because was relevant to SP 2.0 | High level leaders: Motivation | The individual(s) is committed to fulfilling Role. | The individual is motivated to refer to the SP 2.0 program |
| Yes | B. Mid-level Leaders | Individuals with a moderate level of authority, including leaders supervised by a high-level leader who supervise others. | Service Line and/or Supervisor. General feedback about the Clinical coordinators or team leads Note: if the participant is in this role or speaking about others |
| Yes | Mid level leaders: Capability | The individual(s) has interpersonal competence, knowledge, and skills to fulfill Role (*of referral to SP 2.0*). | The individual has sufficient understanding of SP 2.0 to effectively educate staff and support staff to refer to SP 2.0 as well as refer to SP 2.0 themselves. |
| Yes | Mid level leaders: Opportunity | The individual(s) has availability, scope, and power to fulfill Role (*of referral to SP 2.0*). | The informant has the time, autonomy, and power to perform their role in supporting SP 2.0 education, referral, and follow-up. |
| No, added in because was relevant to SP 2.0 | Mid level leaders: Motivation | The individual(s) is committed to fulfilling Role. | The informant is motivated to refer the SP 2.0 program |
| No, added in because was relevant to SP 2.0 | H. Innovation Deliverer | Individuals who are directly or indirectly delivering the innovation | Clinicians who refer to SP 2.0 program. Clinicians who are directly or indirectly refer to the SP 2.0 program. |
| Yes | Deliverer: Capability | The individual(s) has interpersonal competence, knowledge, and skills to fulfill Role (*of referral to SP 2.0*). | The individual has sufficient understanding of SP 2.0 to effectively educate staff and support staff to refer to SP 2.0 as well as refer to SP 2.0 themselves. |
| Yes | Deliverer: Opportunity | The individual(s) has availability, scope, and power to fulfill Role (*of referral to SP 2.0*). | The informant has the time, autonomy, and power to perform their role in supporting SP 2.0 education, referral, and follow-up. |
| No, added in because was relevant to SP 2.0 | Deliverer: Motivation | The individual(s) is committed to fulfilling Role. | The informant is motivated to refer the SP 2.0 program |
| No, added in because was relevant to SP 2.0 | I. Innovation Recipients | Individuals who are directly or indirectly receiving the innovation. | Veterans at high risk for suicide. Informants' perceptions of Veterans who are at high risk for suicide and have been referred to/eligible for SP 2.0 |
| No, added in because was relevant to SP 2.0 | Innovation Recipients: Capability | The individual(s) has interpersonal competence, knowledge, and skills to fulfill Role (*of participating in SP 2.0*). | The Veteran has that ability and resources to participate in the virtual SP 2.0 program |
| No, added in because was relevant to SP 2.0 | Innovation Recipient: Opportunity | The individual(s) has availability, scope, and power to fulfill Role (*of referral to SP 2.0*). | The Veteran has the availability to participate in the virtual SP 2.0 program |
| No, added in because was relevant to SP 2.0 | Innovation Recipient: Motivation | The individual(s) is committed to fulfilling Role. | The Veteran has the desire to participate and continue to participate in the virtual SP 2.0 program |
| No, added in because was relevant to SP 2.0 | G. Other Implementation Support | Individuals who support the Implementation Leads and/or Implementation Team Members to implement the innovation. | Suicide Prevention Coordinators support the implementation of SP 2.0 through education about the referral process as well as providing referrals themselves |
|  | **CHARACTERISTICS SUBDOMAIN** |  |  |
|  | **V. IMPLEMENTATION PROCESS DOMAIN** | Define the Implementation Process: Suicide prevention team, All Mental health Clinics, Emergency Care |  |
| Yes | H2. Reflecting & Evaluating: Innovation | Collect and discuss quantitative and qualitative information that indicates the degree to which innovation outcomes are achieved. | There is tracking of eligible Veterans for the SP 2.0 program as well as Veterans who have recently completed the program or dropped out so there is not a gap in care. |
| Excluded because not relevant to SP 2.0 | ~~Culture Recipient Centeredness~~ |  |  |
| Excluded because not relevant to SP 2.0 | ~~Available Resources: Space~~ |  |  |
| Excluded because not relevant to SP 2.0 | ~~Structural Characteristics: Physical Infrastructure~~ |  |  |
| Excluded because not relevant to SP 2.0 | ~~Available Resources: Materials & Equipment~~ |  |  |

Coding Notes:

- "The Thing" is SP 2.0 referrals
- If there is a suggested facilitator put "suggested" in front of the code.
- Implementation Support: Suicide Prevention Coordinators are implementation support (they do not deliver the innovation of SP 2.0),
- Implementation Team: Implementation team is SP 2.0 staff.
